# Energetic Origins of the Hydrogen-Bond Redshift: IQA Partitioning of Normal Mode Force Constants

**DOI:** 10.1021/acs.jpca.5c02940

**Published:** 2025-07-07

**Authors:** Leonardo J. Duarte, Erick H. S. Alves, Ataualpa A. C. Braga

**Affiliations:** Departamento de Química Fundamental, 28132 Instituto de Química, Universidade de São Paulo, Avenida Professor Lineu Prestes, 748, São Paulo 05508-000, Brazil

## Abstract

The redshift of the D–H (where D represents the H donor group containing F, O, N) bond stretch normal coordinate is notable evidence of a hydrogen-bonded system. Upon the formation of the hydrogen bond, electron density is transferred from the acceptor moiety of the complex to the hydrogen atom donor, causing an elongation of the D–H bond and a reduction of the force constant. Within the orbital paradigm of chemistry, the redshift of the D–H stretch frequency is caused by the donation of electrons from the base lone pair to the antibonding, σ*, orbital of the D–H bond. The increased electron population of the antibonding orbital is, therefore, responsible for the decrease in the force constant and, consequently, for the redshift. In this work, we present a description of the H-bond redshift in terms of the electron density, substituting the molecular orbital theory interpretation with the Quantum Theory of Atoms in Molecules with the Interacting Quantum Atoms (IQA) energy decomposition scheme. The results herein suggest that the energetic origin of the redshift depends on the acid structure. When H_2_O acts as a H donor, the redshift is mostly determined by the intratomic and exchange–correlation, whereas the redshift of the HF stretch is caused primarily by the Coulomb contribution. The H acceptor molecule modulates the amount of variation in the above-mentioned cases. The better the acceptor, the greater the variation in the IQA contribution to the force constant.

## Graphical abstract

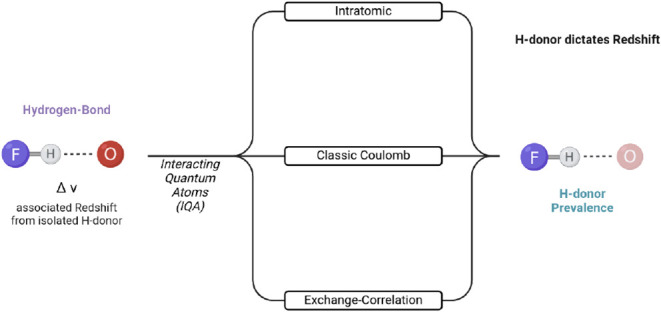

## Introduction

The hydrogen bond is one of the most important chemical interactions, being responsible for various physical properties of compounds, from p*K*_a_ ^1^ to boiling points,^2^ crystal structures,^3^ and biological processes.^4,5^ Due to its importance in many fields, numerous articles and books have been dedicated to reporting experimental data and discussing the classification and mechanism of hydrogen-bond formation^6–10^ and their effects on infrared spectra.^11,12^

Perhaps the most known book on the subject is *“The Hydrogen Bond”* by Pimentel and McClellan,^10^ where the H bond is defined as an associative interaction with energy of a few kilojoules per mol responsible for forming a cluster of molecules, called “complexes”. Such interaction exists if

1. there is evidence of a chemical bond
2. such a bond involves a H atom bonded to another atom.

The classification of the hydrogen bond also depends on the nature of the donor and the acceptor.^8,13^ As described in IUPAC’s Gold Book,^14^ the hydrogen in the acid must be bonded to an atom of high electronegativity, usually F, O, or N, and the acceptor must be able to donate electron density, usually by having lone pairs of electrons, to the σ* orbital of the D–H bond. The formation of the hydrogen bond causes changes in the electron density of both the hydrogen donor and acceptor that are probed by two main spectroscopic observations: the redshift and the intensification of the D–H stretch band. It is important to mention that there is also a special class of H bonds where the donor atom or group is not electronegative and/or the acceptor is not a good electron donor group. Such a class of H-bond is called an “improper hydrogen bond”, and its formation leads to an increase in the D–H stretch frequency, a blueshift, and to a decrease in the band intensity. In this paper, however, we focus our attention on the conventional H-bond and its redshift.

Inspired by experimental measurements compiled by Iogansen^15^ and using the Charge–Charge Transfer–Dipolar Polarization (CCTDP)^16^ intensity decomposition scheme, the electronic effects causing the intensification of the D–H stretch band were properly described.^12^ It was found that the intensification results from charge transfer from the acceptor to the donor molecule and from the polarization of the atomic basins due to the reorganization of the electron density. The redshift, on the other hand, is fundamentally caused by a decrease in the force constant of the D–H bond and, therefore, has its origins not directly related to the distribution of the electron density but to how the components of the total energy change when the H bond is formed. The redshift is quantified as the difference between the complex D–H stretch frequency and the corresponding normal mode of the free D–H molecule, that is,

1 $$\Delta \mathrm{\nu ̃}={\mathrm{\nu ̃}}_{\mathrm{DH}}^{\mathrm{Hbond}}-{\mathrm{\nu ̃}}_{\mathrm{DH}}^{\mathrm{free}}$$

where ν̃_DH_^free^ is the frequency of the D–H stretch in the isolated molecule and *ν̃*_DH_^H bonded^ is the frequency of the corresponding normal mode in the H-bond complex. This metric is important since it is correlated with bond length,^17^ enthalpy,^18^ and band intensification of the hydrogen bond. However, since the frequency is proportional to the square root of the force constant, it is convenient, to our approach, to quantify the redshift as the difference between the squared frequencies, that is,

2 $$\Delta \left({\mathrm{\nu ̃}}^{2}\right)={\left({\mathrm{\nu ̃}}_{\mathrm{DH}}^{\mathrm{Hbond}}\right)}^{2}-{\left({\mathrm{\nu ̃}}_{\mathrm{DH}}^{\mathrm{free}}\right)}^{2}$$

which is proportional to the difference between the mass-weighted force constant of the A–H stretch, Δ­(*ν̃* ^2^) ∝ *k*_DH_^H bond^ – *k*_DH_^free^.

The force constant of a chemical bond is the second derivative of the electronic energy with respect to the bond length. In the case of the hydrogen stretch, the force constant can also be obtained as a function of the normal coordinate of vibration since the hydrogen atom, due to its small mass, is essentially the only atom moving in this coordinate of vibration. A method for decomposing force constants in internal coordinates in its IQA components was presented in a previous publication.^19^ Herein, we adopt the same methodology but do the appropriate coordinate transformation to decompose the force constant in terms of the mass-weighted normal coordinates, decomposing the redshift as expressed in eq 2.

## Methodology

Using the topological features of the Quantum Theory of Atoms in Molecules (QTAIM),^20^ the electron density can be divided into atomic domains, from which atomic properties can be calculated. QTAIM is at the core of many methodologies involving the analysis of the electron density, including the Interacting Quantum Atoms (IQA) energy decomposition scheme.^21^ With the formalism of IQA,^22–24^ one can write the electronic energy as

3 $$E_{\mathrm{Total}}=\sum_{A=1}^{N}E_{\mathrm{Intra}}^{A}+\sum_{A=1}^{N-1}\sum_{B>A}^{N}V_{\mathrm{cl}}^{\mathrm{AB}}+\sum_{A=1}^{N-1}\sum_{B>A}^{N}V_{\mathrm{xc}}^{\mathrm{AB}}$$

where *N* is the number of atoms in the system, *E*_intra_^A^ is the sum of the kinetic energy and Coulomb potential calculated within the atomic basin of atom *A*, *V*_*cl*_^AB^ and *V*_*xc*_^AB^ are, respectively, the classical potential and the exchange–correlation energy between atoms A and B. While the intratomic contributions are related to charge transfer and steric effects,^25,26^ the classical potential is related to the ionicity of a chemical bond, and exchange–correlation contribution can be associated with covalency and hyperconjugation.^27^

In the same way as the total energy, its derivative can also be decomposed into atomic contributions. In particular, the second derivative of the total energy with respect to the Cartesian coordinates *x*_*i*_ and *x*_*j*_ is given by

4 $$\frac{\partial^{2}E_{\mathrm{total}}}{\partial x_{i}\partial x_{j}}=\sum_{A=1}^{N}\frac{\partial^{2}E_{\mathrm{Intra}}^{A}}{\partial x_{i}\partial x_{j}}+\sum_{A=1}^{N-1}\sum_{B>A}^{N}\frac{\partial^{2}V_{\mathrm{cl}}^{\mathrm{AB}}}{\partial x_{i}\partial x_{j}}+\sum_{A=1}^{N-1}\sum_{B>A}^{N}\frac{\partial^{2}V_{\mathrm{xc}}^{\mathrm{AB}}}{\partial x_{i}\partial x_{j}}$$

The derivative on the LHS of eq 4 is the *f*_*ij*_^(*X*)^ element of the Hessian matrix, **F** ^(**X**)^, *i.e*., it corresponds to the force constant with respect to displacements in the *x*_*i*_ *x*_*j*_ direction. The IQA derivatives can also be written in the form of a 3*N* × 3*N* square matrix, that is,

5 $$F^{\left(x\right)}=\sum_{A=1}^{N}F_{\mathrm{Intra}}^{\left(x\right)A}+\sum_{A=1}^{N-1}\sum_{B>A}^{N}F_{\mathrm{cl}}^{\left(x\right)\mathrm{AB}}+\sum_{A=1}^{N-1}\sum_{B>A}^{N}F_{\mathrm{xc}}^{\left(x\right)\mathrm{AB}}$$

Notice that eq 5 decomposes each element of the Hessian matrix into IQA components.

The vectors of the normal modes of vibration are found by diagonalization of **F** ^(**X**)^ **L**, which is done by solving the eigenvalue problem^28^

6 $$F^{\left(X\right)}L=\mathrm{ML}\Lambda$$

where **M** is the diagonal matrix of atomic masses. **Λ** is the diagonal eigenvalue matrix and **L** is the matrix containing the normal coordinates of each molecular vibrational mode expressed in the Cartesian basis. Since the Hessian matrix is a sum of IQA matrices, the same can be said about the eigenvalues. Substituting eq 5 into eq 6, one has

7 $$\sum_{A=1}^{N}F_{\mathrm{Intra}}^{\left(x\right)A}L+\sum_{A=1}^{N-1}\sum_{B>A}^{N}F_{\mathrm{cl}}^{\left(x\right)\mathrm{AB}}L+\sum_{A=1}^{N-1}\sum_{B>A}^{N}F_{\mathrm{xc}}^{\left(x\right)\mathrm{AB}}L=\mathrm{ML}\left(\sum_{A=1}^{N}\Lambda_{\mathrm{Intra}}+\sum_{A=1}^{N-1}\sum_{B>A}^{N}\Lambda_{\mathrm{cl}}+\sum_{A=1}^{N-1}\sum_{B>A}^{N}\Lambda_{\mathrm{xc}}\right)$$

Each one of the **Λ**_**Intra**_, **Λ**_**cl**_, and **Λ**_**xc**_ contains, respectively, the intratomic, Coulomb, and exchange–correlation atomic contributions to the eigenvalue of each normal mode. Alternatively, one can write

8 $$L^{†}F^{\left(X\right)}L=F^{\left(Q\right)}$$

with **F** ^**Q**^ being a diagonal matrix containing the force constant of each normal mode. Notice that **F** ^**Q**^ can also be decomposed into IQA contributions

9 $$F^{\left(Q\right)}=\sum_{A=1}^{N}F_{\mathrm{Intra}}^{\left(Q\right)A}+\sum_{A=1}^{N-1}\sum_{B>A}^{N}F_{\mathrm{cl}}^{\left(Q\right)\mathrm{AB}}+\sum_{A=1}^{N-1}\sum_{B>A}^{N}F_{\mathrm{xc}}^{\left(Q\right)\mathrm{AB}}$$

where the diagonal elements of **F**_**Intra**_^(**Q**)**A**^, **F**_**cl**_^(**Q**)**AB**^, and **F**_**xc**_^(**Q**)**AB**^ are the IQA force constant contributions to the normal modes of vibration. The off-diagonal elements from those matrices are not considered physically relevant. Notice that, as **F** ^(**Q**)^ is diagonal, the sum of the off-diagonal elements of **F**_**Intra**_^(**Q**)**A**^, **F**_**cl**_^(**Q**)**AB**^, and **F**_**xc**_^(**Q**)**AB**^ is always zero.^19^

Since no analytical methods are available for the calculation of the IQA contributions to *F* ^(*X*)^, the numerical derivation of the IQA terms requires at least 18N^2^ single-point calculations; therefore, we limit this study to small H-bonded complexes.

## Computational Details

All molecules and complexes had their geometries optimized at the B3LYP/aug-cc-pVTZ levels of theory using GAUSSIAN16 software.^29^ The wave function of each system was integrated using AIMAll software,^30^ yielding the QTAIM/IQA parameters needed to compute the force constants. The use of the B3LYP functional is justified since the calculation of the IQA terms is currently restricted to a few functionals^23^ [Fn fn1].

Numerical differentiation is utilized to compute the infrared frequencies of molecules and complexes. Starting from the equilibrium geometry of the system, distorted geometries are generated by displacing each atom in the positive and negative directions of each Cartesian axis. For each geometry, the IQA components are obtained. The data recovered from the calculations are used to calculate numerical second derivatives and construct the Hessian matrix, **F** ^(**X**)^ **L**.

The vibrational frequencies are determined by diagonalizing the mass-weighted Hessian. IQA contributions to the Hessian eigenvalues, i.e., the force constants, are also calculated. The procedure can be automated using the Theovib Python library available at github.com/ljduarte/theovib.

## Results and Discussion

### Decomposition of Force Constants

The H-bond complexes having HF, H_2_O, or NH_3_ acting as hydrogen donors, i.e., the acid moiety, and HCN, NH_3_, H_2_CO, and CH_3_OH are the set of hydrogen-bond acceptors; hence, the basis had their geometry optimized, and normal modes were calculated using the numerical approach and the aforementioned IQA contributions. In order to simplify the analysis and reduce the number of terms, the IQA contributions to the force constant are grouped as follows

10 $${Ë}_{\mathrm{Intra}}^{\mathrm{Acid}}=\sum_{I\in \mathrm{Acid}}{Ë}_{\mathrm{Intra}}^{I}$$

11 $${Ë}_{\mathrm{Intra}}^{\mathrm{Base}}=\sum_{I\in \mathrm{Base}}{Ë}_{\mathrm{Intra}}^{I}$$

12 $${\mathrm{V̈}}_{\mathrm{cl}}^{\mathrm{aa}}=\frac{1}{2}\sum_{I\in \mathrm{Acid}}\sum_{J\in \mathrm{Acid}}{\mathrm{V̈}}_{\mathrm{cl}}^{\mathrm{IJ}}$$

13 $${\mathrm{V̈}}_{\mathrm{cl}}^{\mathrm{ab}}=\sum_{I\in \mathrm{Acid}}\sum_{J\in \mathrm{Base}}{\mathrm{V̈}}_{\mathrm{cl}}^{\mathrm{IJ}}$$

14 $${\mathrm{V̈}}_{\mathrm{cl}}^{\mathrm{bb}}=\frac{1}{2}\sum_{I\in \mathrm{Base}}\sum_{J\in \mathrm{Base}}{\mathrm{V̈}}_{\mathrm{cl}}^{\mathrm{IJ}}$$

15 $${\mathrm{V̈}}_{\mathrm{xc}}^{\mathrm{aa}}=\frac{1}{2}\sum_{I\in \mathrm{Acid}}\sum_{J\in \mathrm{Acid}}{\mathrm{V̈}}_{\mathrm{xc}}^{\mathrm{IJ}}$$

16 $${\mathrm{V̈}}_{\mathrm{xc}}^{\mathrm{ab}}=\sum_{I\in \mathrm{Acid}}\sum_{J\in \mathrm{Base}}{\mathrm{V̈}}_{\mathrm{xc}}^{\mathrm{IJ}}$$

17 $${\mathrm{V̈}}_{\mathrm{xc}}^{\mathrm{bb}}=\frac{1}{2}\sum_{I\in \mathrm{Base}}\sum_{J\in \mathrm{Base}}{\mathrm{V̈}}_{\mathrm{xc}}^{\mathrm{IJ}}$$

Notice that the contributions to the force constants are second derivatives of the IQA energy terms, as shown in eq 4. In order to clarify the notation, we use Newton’s notation for differentiation, that is $Ë=\frac{d^{2}E}{{dQ}^{2}}$ . *Ë*_Intra_^Acid^ contains only intratomic contributions from the acid, that is, the hydrogen donor, and *Ë*_Intra_^Base^ contains only the contributions from the base, i.e., the hydrogen-bond acceptor. Similarly, *V̈*_*cl*_^aa^ is the sum of all classical Coulomb contributions between atoms from the acid, *V̈*_*cl*_^ab^ gathers all classical Coulomb contributions between atoms from the acid and the base, and *V̈*_*cl*_^bb^ is the resulting classical Coulomb contribution between atoms from the base. The same applies to the exchange–correlation terms.

Table 1 shows the D–H stretch frequency for the H-bond complexes and for the three acids analytically calculated by GAUSSIAN16 and numerically calculated using the IQA contributions. The root mean squared difference (RMSD) between the analytical and IQA frequencies is 25.84 cm^–1^, or 0.72% of the average frequency, indicating low numerical errors. The parameters utilized in the computation of the frequencies are the same as described in the original methodology paper.^19^

**Table 1:** Analytical Frequency of the A–H Stretch Calculated by GAUSSIAN16, in cm^–1^, Numerical Frequency Calculated Using IQA Contributions, in cm^–1^ and IQA Contributions to the Force Constant, in Hartree ·Å^–2^ · amu^–1^ ^a^

acid	base	*ν̃*_Gaussian_	*ν̃*_IQA_	error	*Ë*_Intra_^Acid^	*Ë*_Intra_^Base^	*V̈*_*cl*_^aa^	*V̈*_*cl*_^ab^	*V̈*_*cl*_^bb^	*V̈*_*xc*_^aa^	*V̈*_*xc*_^ab^	*V̈*_*xc*_^bb^
H_2_O	HCN	3736.57	3755.57	19.00	0.14	0.04	0.46	–0.06	–0.01	–0.07	–0.02	0.01
H_2_O	NH_3_	3535.22	3549.87	14.65	0.07	0.04	0.50	–0.01	–0.01	–0.07	–0.05	0.03
H_2_O	H_2_CO	3680.83	3696.8	15.97	0.14	–0.01	0.46	0.00	0.01	–0.07	–0.03	0.01
H_2_O	CH_3_OH	3644.07	3650.53	6.46	0.10	0.02	0.50	–0.02	–0.04	–0.08	–0.04	0.02
HF	HCN	3742.46	3780.09	37.63	–0.35	0.08	1.06	–0.01	–0.03	–0.18	–0.05	0.02
HF	NH_3_	3214.42	3198.02	16.40	–0.41	0.05	0.96	–0.04	–0.03	–0.15	–0.09	0.03
HF	H_2_CO	3610.96	3619.01	8.05	–0.37	0.03	1.05	–0.02	0.03	–0.17	–0.06	0.02
HF	CH_3_OH	3516.85	3586.21	69.36	–0.37	0.05	1.03	–0.02	–0.06	–0.16	–0.07	0.03
NH_3_	HCN	3463.45	3471.33	7.88	0.40	0.01	0.03	0.00	0.00	0.03	–0.01	0.00
NH_3_	NH_3_	3426.76	3446.49	19.73	0.43	0.02	–0.03	0.00	–0.01	0.05	–0.02	0.01
NH_3_	H_2_CO	3454.19	3474.67	20.48	0.42	0.00	0.00	0.00	0.00	0.03	–0.01	0.00
monomers												
HF	—	4072.01	4101.27	29.26	–0.28	—	1.16	—	—	–0.24	—	—
H_2_O	—	3797.81	3811.4	13.59	0.38	—	0.19	—	—	–0.02	—	—
NH_3_	—	3468.17	3470.21	2.04	0.40	—	0.04	—	—	0.02	—	—

a The column “error” corresponds to the absolute error between the IQA and Gaussian frequencies

The contributions from atoms belonging to the acid moiety of the H-bond complex are more pronounced than contributions from the base’s atoms. Moreover, contributions from intermolecular interactions, either *V̈*_*cl*_^ab^ or *V̈*_*xc*_^ab^, are surprisingly low. This suggests that the force constant of the D–H stretch is not directly influenced by contributions from the base. The formation of the hydrogen bond, however, modifies the acid’s electron density, causing a decrease in the force constant.

Since contributions from the base are negligible, we look only at the variations from the acid contributions. Table 2 shows the variation of the acid contributions, the red-shift, and the hydrogen-bond formation enthalpy. It is well-known that there is a linear correlation between enthalpy and Δ­(*ν̃* ^2^). In the left panel of Figure 1, it is shown that the correlation between enthalpy and red-shift is conserved if the difference of squared frequencies is utilized. As a matter of fact, the squared correlation coefficient between the quantities is 0.976.

**Figure 1:**
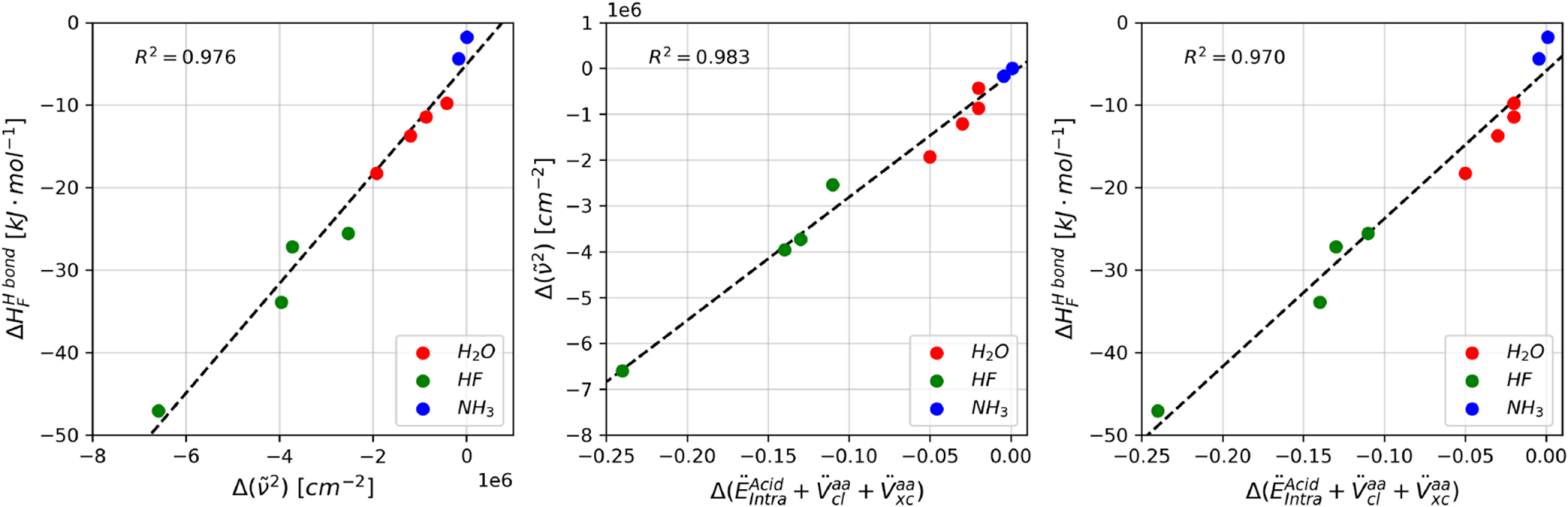
Linear correlation between Hydrogen-bond enthalpy of formation and redshift (left panel), redshift and sum of acid contributions to the force constant (middle panel), and between the sum of the IQA contributions and the enthalpy of formation (right panel). The dotted line is the regression line between the quantities.

**Table 2:** Redshift, in cm^–2^, Variation of the Acid IQA Contributions to the A–X Stretch Force Constant, in Hartree ·Å^–2^ ·amu^–1^, and Hydrogen-Bond Formation Enthalpy, in kJ · mol^–1^

acid	base	Δ(*ν̃* ^2^)	Δ*Ë*_Intra_^Acid^	Δ*V̈*_*cl*_^aa^	Δ*V̈*_*xc*_^aa^	Δ(*Ë*_Intra_^Acid^ + *V̈*_*cl*_^aa^ + *V̈*_*xc*_^aa^)	Δ*H*_*F*_^H bond^
H_2_O	HCN	–422463.94	–0.24	0.27	–0.05	–0.02	–9.76
H_2_O	NH_3_	–1925192.94	–0.31	0.31	–0.05	–0.05	–18.28
H_2_O	H_2_CO	–860439.72	–0.24	0.27	–0.05	–0.02	–11.39
H_2_O	CH_3_OH	–1200400.68	–0.28	0.31	–0.06	–0.03	–13.71
HF	HCN	–2531335.20	–0.07	–0.10	0.06	–0.11	–25.55
HF	NH_3_	–6593083.69	–0.13	–0.20	0.09	–0.24	–47.05
HF	H_2_CO	–3723182.23	–0.09	–0.11	0.07	–0.13	–27.13
HF	CH_3_OH	–3959513.45	–0.09	–0.13	0.08	–0.14	–33.87
NH_3_	HCN	7774.52	0.01	–0.01	0.00	0.00	–1.76
NH_3_	NH_3_	–164064.12	0.04	–0.07	0.02	0.00	–4.38
NH_3_	H_2_CO	30974.16	0.02	–0.03	0.01	0.00	–4.80

Neglecting the contribution from the base, we also find a correlation between the red-shift and the variation of the acid contributions, *R* ^2^ = 0.983. This is interesting since it reveals that the basis modifies the acid electron density upon the formation of the hydrogen bond, but the energetic factors contributing to reducing the frequency of the D–H stretch originate within the H donor molecule. Such factors are also correlated (*R* ^2^ = 0.970) with the enthalpy of formation of the H bond (right panel, Figure 1). Although such a correlation does not imply causality, it suggests a common source for both observations.

The variation in the acid IQA contributions is not the same for all acids. Figure 2 shows the variation in the IQA contributions for each complex. Notice that the changes in the IQA terms derivatives can either be positive or negative. A positive variation indicates that the contribution is increasing the force constant, therefore causing a blueshift in the D–H stretch. On the other hand, the negative contributions are those responsible for the redshift.

**Figure 2:**
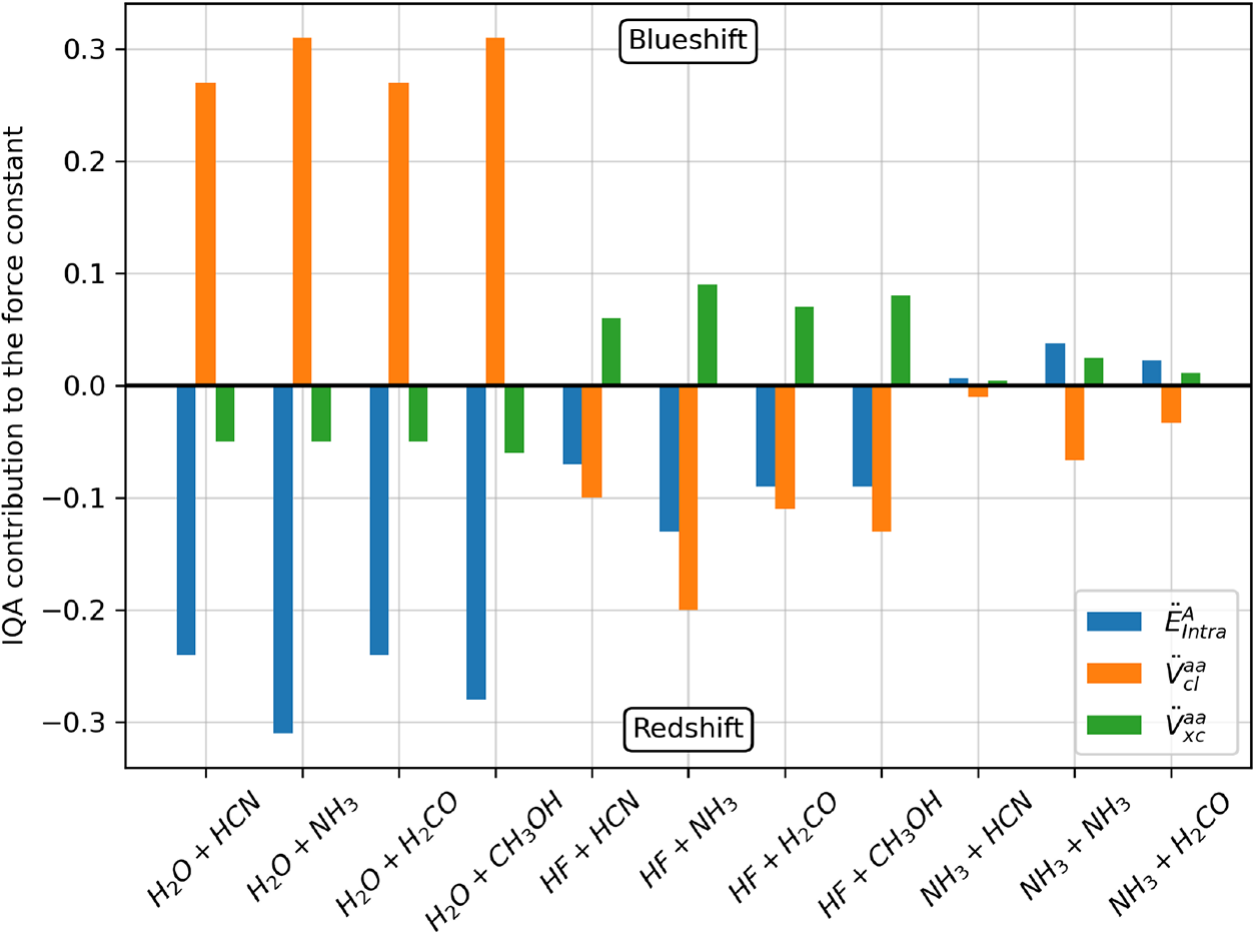
Variation of the IQA contributions to the force constant of D–H stretch. A positive value indicates that the contribution increases the force constant after the formation of the hydrogen bond, and a negative value indicates that the contribution reduces the force constant, producing a redshift.

For H_2_O, the intratomic energy derivatives are the main factor causing the redshift, while the classical Coulomb derivatives increase the force constant. This is not the case for HF, where both the intramolecular and classical Coulomb derivatives contribute to the redshift while the exchange–correlation derivatives increase the force constant. A third case is found for NH_3_, where only classical Coulomb derivatives are negative. The only factor that is similar for the three groups is that the exchange–correlation derivatives are always lower, in absolute value than the intramolecular and Coulomb derivatives. Summing all contributions’ variations and applying the appropriate conversion constants, one will find that the contributions are equivalent to the redshift defined in eq 2.

The stronger the acid, the greater the red-shift and the more stable the hydrogen bond, as shown in Figure 1. NH_3_ is the weakest acid in our data and produces complexes with very low to no redshift, and its IQA derivatives also show smaller variations. HF complexes are the ones with greater redshift, but their IQA derivative variations are smaller than the variations found in H_2_O, as the canceling nature of the IQA variations is greater for the latter than for the former. This suggests that the H_2_O molecule experiences greater perturbation in its electron density when the H bond is formed, but the different signs in the variation lead to a smaller redshift.

Another interesting observation from Figure 2 is that the base does not affect the relative signs of the IQA derivative variations, which seem to depend only on the nature of the acid, but it affects only the magnitude of those variations. The stronger the base, the greater the variations of the IQA derivatives.

### D–H Potential Energy Well

In the chemical literature, force constants are usually taken as a measure of bond stability. As mentioned in a previous publication, such an analysis should be made carefully, as the bond stability is related to the depth of the potential well, while the force constant is related to its curvature at the equilibrium point; that is, it describes the width of the potential well. The correlation between force constant and bond stability only exists if the bonds’ potential energy can be described by a Morse potential and if they share the same constant α that depends on both the force constant *k*_e_ and dissociation energy *D*_e_, as in

18 $$\alpha =\sqrt{\frac{k_{e}}{2D_{e}}.}$$

A bond with high stability in the thermodynamic sense requires a high amount of energy to be broken, but this does not necessarily mean it requires the same amount of energy to be distorted from its equilibrium position. A stable bond can be compliant, thus expressing some “elasticity” and, at the same time, a stiff bond with a high force constant may be easily broken. In this sense, the red-shift indicates that, upon the formation of the hydrogen bond, the H–D bond becomes more elastic and easier to distort.

Figure 3 shows the variation of energy with respect to the D–H bond length for DH···NH_3_. The left panels show the potential well (black line) and the IQA contributions grouped by fragments, i.e., acid, base, and their interactions. Since the variation of curvature of the acid contributions was identified as the main factor for the redshift, the acid contributions are divided into *E*_intra_^Acid^, *V*_*cl*_^aa^, and *V*_*xc*_^aa^ and plotted on the right panels of Figure 3.

**Figure 3:**
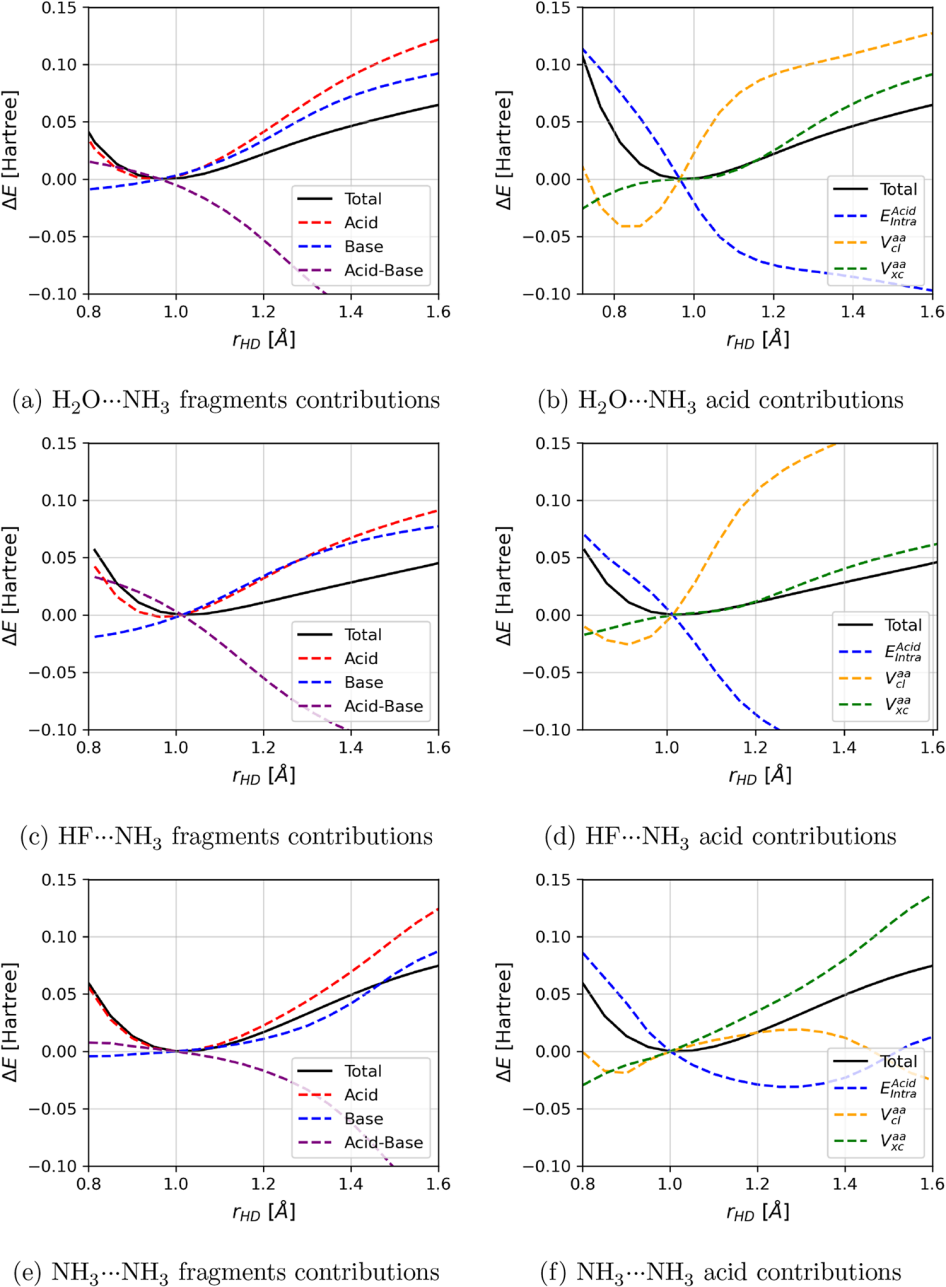
Total electronic energy versus bond distance in DH–NH_3_. Panels on the left show the total IQA contributions for each fragment (acid and base) and their interaction term; on the right, the graph shows the partitioning of the acid contributions. In every plot, at each point, the system geometry is optimized, i.e., the geometry is allowed to relax.

In Figure 3b,3d,3f, there is an inverse correlation between the *V*_*cl*_^aa^ and *E*_Intra_^Acid^. The increase in the Coulomb potential occurs due to the increase in the bond distance, but the intratomic energy is a bit more complicated, as it encompasses not only charge transfer effects but also deformation energy associated with steric hindrance. The complex intratomic energy becomes more negative as the proton is transferred to the base; i.e., the contribution is stabilized by the increase in the electronic population.

The stabilization of the intratomic terms in the acid occurs because the HD stretch coordinate is coupled to the proton transfer reaction coordinate, as in HD + NH_3_ → D^–^ + NH_4_^+^. At the equilibrium geometry, the hydrogen atom has a positive charge and plays an important role in the electron transference channel from the base to the acid. As the H–D bond stretches, the H atom is captured by the base, thus receiving part of the base’s electrons while leaving the transferred electrons at the acid; therefore, we observe a stabilization of *E*_Intra_^Acid^ and, at the same time, a destabilization of *V*_*cl*_^aa^.

As the proton approaches the base, there is a compensation in energy by the stabilization interaction between the base and the proton, shown by the purple curve on the left panels of Figure 3, but such stabilization is relevant far from the equilibrium point. The presence of the base makes the H–D bond easier to break, but the curvature of the well is still mostly determined by the acid contributions, as shown by the force constant decomposition.

The role of the hydrogen atom as a charge transfer channel is probed as its atomic charge increases upon the formation of the H bond. Table 3 contains the total AIM atomic charges for the acid in the complexes, as well as in its free form. As the charges are conserved in the system, the complex acid charge is also the total transferred charge upon the formation of the hydrogen bond.

**Table 3:** AIM Atomic Charges [in *e*] for the H-Bond Hydrogen (*q*_*H*_), Sum of the Atomic Charges of the Other Hydrogen Atoms in D–H (∑*q*_*H*′_), Total Transferred Charge (Δ*q*_Acid_) upon the Formation of the Hydrogen Bond and Charge Variation of the H-Bond Hydrogen Atom (Δ*q*_*H*_)

acid	base	*q*_*H*_	∑*q*_*H*′_	*q*_*D*_	Δ*q*_Acid_	Δ*q*_*H*_
HF	HCN	0.743	—	–0.779	–0.037	0.013
HF	NH_3_	0.723	—	–0.804	–0.080	–0.006
HF	H_2_CO	0.741	—	–0.781	–0.041	0.011
HF	CH_3_OH	0.743	—	–0.790	–0.047	0.014
H_2_O	HCN	0.603	0.560	–1.180	–0.017	0.031
H_2_O	NH_3_	0.608	0.554	–1.205	–0.043	0.036
H_2_O	H_2_CO	0.604	0.567	–1.190	–0.019	0.032
H_2_O	CH_3_OH	0.611	0.560	–1.194	–0.024	0.039
NH_3_	HCN	0.379	0.652	–1.051	–0.020	0.041
NH_3_	NH_3_	0.369	0.658	–1.034	–0.006	0.031
NH_3_	H_2_CO	0.369	0.670	–1.039	0.001	0.031
monomers						
HF	—	0.729	0.000	–0.729	—	—
H_2_O	—	0.572	0.572	–1.144	—	—
NH_3_	—	0.338	0.677	–1.015	—	—

The hydrogen atom becomes more positive once the interaction with the base is established. The overall acid charge is, however, negative, thus indicating that the H atom acts as a channel for transferring electrons from the Lewis base to the acid. This transference of electrons is already known for being the main reason behind the infrared intensification of the D–H stretch, as it not only changes the atomic charges but also triggers a chain of atomic dipole moment reorganization throughout the system.

In the case of force constants, the transference of electrons affects the contributions of the acid, changing the curvature of the IQA contributions. How those changes translate into the redshift is, however, dependent on the acid, as it is relative to the free acid D–H stretch force constant.

In a very interesting work by Freindorf, Kraka, and Cremer,^11^ it was demonstrated that the redshift of the D–H bond can be related to the local force constant of the newly formed H–A bond, with A being the base or the H-bond acceptor. This is interesting since, different from the force constant decomposition presented herein, local modes are localized in a specific bond. Therefore, while we have shown that the origin of the redshift depends on the nature of the acid, its magnitude depends on the base. The local mode analysis of the HA bond, therefore, is able to encompass both effects, resulting in a quantitative measure of the H-Bond strength.

## Conclusions

A description of the H-bond red-shift, discerning the contributing factors in the total energy utilizing the IQA energy decomposition scheme, grounded by the QTAIM approach, is presented in this work. The results suggest that the contributing distribution is mostly dependent on the acid moiety, while its magnitude is modulated by the base strength. The intermolecular (acid–base) contributions, either Coulombic and exchange–correlation, are close to negligible, indicating little dependence of the force constants on these terms. The acid contribution variation shows an excellent correlation between the squared redshift and the bond’s enthalpy of formation, demonstrating its direct impact on the D–H bond stretch frequency. Apart from the whole quantity, if decomposed, we can see that a greater perturbation in the electronic distribution does not imply a greater redshift once the canceling factors decide the magnitude of the latter. Lastly, an energetic analysis of the bond distortion around its equilibrium structure showed that the Coulombic potential becomes relevant far from the equilibrium structure in an established H bond. During the H-bond formation, the H atom acts as a charge bridge between the base electrons and the acid moiety. The IQA force constant decomposition scheme served as a key to getting insights into the origins and aspects of the H-bonded system.

## Supplementary Material



## Footnotes

1 Currently, the following functionals are supported by AIMAll: LSDA, B3LYP, M062X, PBE, and PBE0

## Notes

Molecular geometries are available in the Supporting Information file. The Theovib library is open-source and can be downloaded at github.com/ljduarte/theovib or directly installed from the Python Package Index ($pip install theovib) (PDF)
