# Supplementary material for: Energetic Origins of the Hydrogen-Bond Redshift: IQA Partitioning of Normal Mode Force Constants
Source: J Phys Chem A. 2025 Jul 7;129(28):6281–8. doi: 10.1021/acs.jpca.5c02940 (PMC12278215; doi:10.1021/acs.jpca.5c02940)
Supplement: Supplementary file 1 [file jp5c02940_si_001.pdf]

Supplementary information

Energetic origins of the hydrogen-bond redshift: IQA partitioning of normal modes force constants

Leonardo J. Duarte[1*], Erick H. S. Alves[1], Ataualpa A. C. Braga[1]

[1] Departament of Fundamental Chemistry, Institute of Chemistry, University of São Paulo, Av. Prof. Lineu Prestes, 748, São Paulo, 05508-000, São Paulo, Brazil.

*Corresponding author(s). E-mail(s): ljduarte@iq.usp.br;

Keywords: IR, Force constants, Redshift, Hydrogen bond

1 Cartesian Coordinates of Optimized Structures

Table S1: Optimized structure of $H_2O \cdots HCN$.

Atom	X	Y	Z
C	0.00430600	-0.00012200	-0.72231600
H	0.13652700	-0.00030500	-1.78024600
N	-0.13630100	0.00007500	0.41335400
H	-0.07127300	0.00067100	2.53469800
O	0.02909400	0.00099000	3.49617400
H	-0.86728600	0.00044600	3.84219000

Table S2: Optimized structure of $H_2O \cdots NH_3$.

Atom	X	Y	Z
H	-2.67373600	-1.26692500	-1.60343000
N	-2.18892800	-0.84818700	0.25856000
O	-2.84685100	-1.61027000	-2.50110400
H	-1.53913600	-0.07943000	0.37435700
H	-2.96928700	-0.68497700	0.88388100
H	-1.72025200	-1.68987900	0.57374200
H	-3.31221600	-0.90801300	-2.96247300

Table S3: Optimized structure of $H_2O \cdots H_2CO$.

Atom	X	Y	Z
O	-0.76658400	0.55066100	0.28090300
C	-0.52355400	0.11636800	-0.81620600
H	-1.22833900	0.24865000	-1.65450700
H	0.41026700	-0.42975400	-1.02808400
H	0.78364900	0.05888300	1.40809400
O	1.64941100	-0.34894100	1.56233800
H	1.84886500	-0.19411200	2.48915200

Table S4: Optimized structure of $H_2O \cdots CH_3OH$.

Atom	X	Y	Z
C	0.27620700	0.84832700	-0.07639600
O	0.35677000	-0.54286200	-0.39871300
H	-0.67561800	1.20324700	-0.46338000
H	0.29971300	1.01359100	1.00289100
H	1.08245200	1.41248300	-0.55015300
H	1.18441800	-0.89865700	-0.06314500
H	-1.12210400	-1.62074400	0.16451500
O	-1.85167400	-2.12594000	0.55927400
H	-2.27924100	-2.57414800	-0.17506800

Table S5: Optimized structure of $HF \cdots HCN$.

Atom	X	Y	Z
C	0.00000000	0.00000000	-0.56788900
H	0.00000000	0.00000000	-1.63438300
N	0.00000000	0.00000000	0.57472900
H	0.00000000	0.00000000	2.39707600
F	0.00000000	0.00000000	3.33596700

Table S6: Optimized structure of $HF \cdots NH_3$.

Atom	X	Y	Z
H	-1.53641600	-0.45669500	0.00094800
N	-0.11225200	0.36089100	0.30799200
H	0.68824300	-0.15704200	-0.03541800
H	-0.10785800	1.26933400	-0.14110800
H	0.01836400	0.50745900	1.30219200
F	-2.35831600	-0.92851600	-0.17625400

Table S7: Optimized structure of $HF \cdots H_2CO$.

Atom	X	Y	Z
O	0.05215800	0.66742800	-0.10091900
C	-0.42587900	-0.13726600	-0.86272300
H	-1.01491600	0.18784000	-1.73365800
H	-0.29271300	-1.22057300	-0.71779100
H	0.93423600	0.00475300	1.19439500
F	1.38372200	-0.51218200	1.84519600

Table S8: Optimized structure of $HF \cdots CH_3OH$.

Atom	X	Y	Z
C	0.26919400	0.83193300	-0.04878700
O	0.29075700	-0.58272200	0.19006600
H	-0.58423800	1.22436600	0.49704800
H	1.17961700	1.30044000	0.32673800
H	0.14733600	1.05507100	-1.10990300
H	1.03346200	-0.97990900	-0.27401700
H	-1.12946100	-1.39081700	-0.05012300
F	-1.95617900	-1.82502900	-0.22102100

Table S9: Optimized structure of $NH_3 \cdots NH_3$.

Atom	X	Y	Z
N	1.615051	0.000032	-0.078246
H	0.901110	-0.000460	0.642763
H	2.198231	0.815061	0.069620
H	2.198656	-0.814819	0.068877
N	-1.615318	0.000037	0.078367
H	-2.198333	-0.814950	-0.070352
H	-0.899493	-0.000255	-0.640795
H	-2.198302	0.814936	-0.070958

Table S10: Optimized structure of $NH_3 \cdots HCN$.

Atom	X	Y	Z
N	-2.416119	-0.003836	-0.126452
H	-1.406163	-0.001634	-0.030607
H	-2.764647	-0.814237	0.371707
H	-2.766362	0.814709	0.357024
C	2.198787	-0.006158	-0.021485
H	3.261826	-0.023522	-0.098283
N	1.056494	0.012640	0.059176

Table S11: Optimized structure of $NH_3 \cdots H_2CO$.

Atom	X	Y	Z
N	1.960919	0.127512	0.000017
H	1.211866	-0.558323	0.000204
H	2.537229	-0.051710	0.813758
H	2.535852	-0.050668	-0.814930
C	-1.349784	0.506326	0.000097
H	-2.384771	0.894231	-0.001278
H	-0.533208	1.247301	0.001445
O	-1.124337	-0.676421	0.000013
